# Evaluation of immunogenicity of *rSeM *and use of PAMPs as possible enhancers of the immune response

**DOI:** 10.1186/1753-6561-8-S4-P161

**Published:** 2014-10-01

**Authors:** Matheus Costa da Rosa, Vitória Sequeira Gonçalves, Itauá Leston Araujo, Alceu Gonçalves dos Santos Junior, Lívia Budziarek Eslabão, Jéssica Rodrigues Orlandin, Renan Araujo Piraine

**Affiliations:** 1 Universidade Federal de Pelotas, Pelotas, RS, Brazil

## 

The immune system is responsible for the first immune response to infection. This response is based, in part, by Toll-Like Receptors (TLR) that detect pathogens and induce an appropriate immune response. These receptors bind to structures called Pathogen Associated Molecular Patterns (PAMPs), including lipopolysaccharide, flagellin, lipoproteins, nucleic acids, and other molecules. The Equine Strangles is a bacterial disease caused by *Streptococcus equi *subspecie *equi *(*S.equi*). This bacterium synthesizes several virulence factors, among these M protein (SeM) that stands out for its high antifagocit potential, having an important role in the pathogenesis, making it a promising antigen vaccine candidate. The purpose of this study was to evaluate the immunogenicity of rSeM and the use of PAMPs as possible potentiators of the immune response.

Forty Balb/c female mice were immunized intramuscularly on day 0 and 21 of the experiment. Blood samples were collected on days 0, 14 and 28, and processed in order to obtain serum. The mice were divided equally into four groups. The animals belong in groups 1-2 were immunized with the recombinant BL21 (DE3) strain of *E.coli*, which was previously cloned to express the protein of interest recombinant M protein (*rSeM*). In group 1, the strain of *E.coli *was inactivated using 0.1% formaldehyde and increased by 10% aluminum hydroxide, and group 2 received the same strain, but was not inactivated and adjuvant was not added in the vaccine composition. Group 3 was immunized only with the recombinant M protein of *rSeM *Purified associated with 10% aluminum hydroxide. Each vaccine dose contains 20 μg of *rSeM*. Animals of to group 4 were used as control and were inoculated with only PBS1X. The immune response of the animals was evaluated by indirect ELISA. It is noteworthy that the animals were kept and handled in accordance with the legal requirements provided for in the Brazilian National Law for Protection of Experimental Animals.

Vaccines containing bacterins, used in group 1 and 2 showed the highest antibody seroconversions compared to the group immunized only with the *rSeM *recombinant vaccine. These results suggest that group 1 and 2 showed higher antibodies absorbance than group 3 due to the fact that these two groups were immunized with the recombinant BL21 (DE3) strain of *E.coli*, which presents the PAMPs.

This work was funded by "Centro de Desenvolvimento Tecnológico (CDTec)" from "Universidade Federal de Pelotas (UFPel)".

